# 3,3-Dimethyl-1,2,3,4,6,11-hexa­hydro­benzo[*d*]naphtho[2,3-*b*]furan-1,6,11-trione

**DOI:** 10.1107/S1600536808021600

**Published:** 2008-07-19

**Authors:** Huayou Hu

**Affiliations:** a Department of Chemistry and Chemical Engineering, Southeast University, Nanjing, People’s Republic of China

## Abstract

In the title compound, C_18_H_14_O_4_, the cyclo­hexene ring adopts a sofa conformation. In the crystalline state, the mol­ecules are linked into a chain by weak inter­molecular C—H⋯O hydrogen bonds.

## Related literature

For related literature, see: Correa & Romo (1966 ▶); Greve & Friedrichsen (2000 ▶); Hirai *et al.* (1999 ▶); Hu *et al.* (2005 ▶); Ito *et al.* (2000 ▶). For related structures, see: Goldstein *et al.* (1975 ▶); Park *et al.* (1992 ▶).
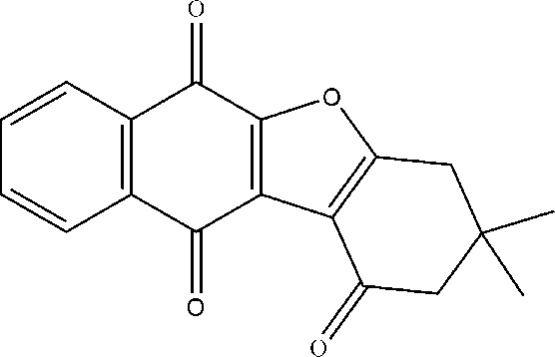


## Experimental

### 

#### Crystal data

C_18_H_14_O_4_
*M* *_r_* = 294.29
Triclinic, 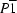

*a* = 5.8080 (12) Å
*b* = 6.7510 (14) Å
*c* = 18.332 (4) Å
α = 89.82 (3)°
β = 81.32 (3)°
γ = 78.18 (3)°
*V* = 695.2 (2) Å^3^
*Z* = 2
Mo *K*α radiation
μ = 0.10 mm^−1^
*T* = 293 (2) K
0.25 × 0.18 × 0.16 mm

#### Data collection

Bruker APEX CCD diffractometer
Absorption correction: multi-scan (*SADABS*; Sheldrick, 2000 ▶) *T*_min_ = 0.976, *T*_max_ = 0.984
2958 measured reflections
2676 independent reflections
1845 reflections with *I* > 2σ(*I*)
*R*_int_ = 0.018

#### Refinement

*R*[*F* ^2^ > 2σ(*F* ^2^)] = 0.047
*wR*(*F* ^2^) = 0.137
*S* = 1.07
2676 reflections
199 parameters
H-atom parameters constrained
Δρ_max_ = 0.16 e Å^−3^
Δρ_min_ = −0.23 e Å^−3^

### 

Data collection: *SMART* (Bruker, 2000 ▶); cell refinement: *SMART*; data reduction: *SAINT* (Bruker, 2000 ▶); program(s) used to solve structure: *SHELXTL* (Sheldrick, 2008 ▶); program(s) used to refine structure: *SHELXTL*; molecular graphics: *SHELXTL*; software used to prepare material for publication: *SHELXTL*.

## Supplementary Material

Crystal structure: contains datablocks I, global. DOI: 10.1107/S1600536808021600/bt2746sup1.cif

Structure factors: contains datablocks I. DOI: 10.1107/S1600536808021600/bt2746Isup2.hkl

Additional supplementary materials:  crystallographic information; 3D view; checkCIF report

## Figures and Tables

**Table 1:** Hydrogen-bond geometry (Å, °)

*D*—H⋯*A*	*D*—H	H⋯*A*	*D*⋯*A*	*D*—H⋯*A*
C16—H16*A*⋯O4^i^	0.93	2.54	3.177 (3)	126

Symmetry code: (i) 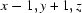.

**Fig. 1.**
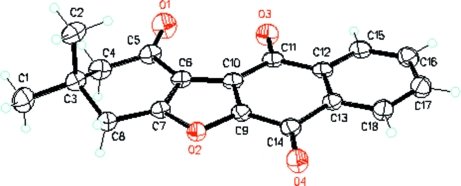
The molecular structure of the title compound with 30% displacement ellipsoids.

**Fig. 2.**
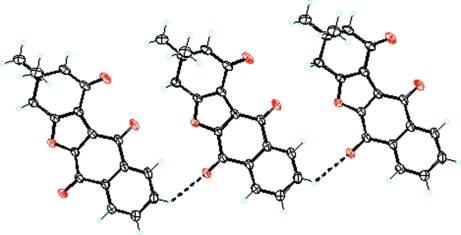
Hydrogen-bonding of the title compound.

## supplementary crystallographic information

### Comment

Furan derivatives and annulated furan derivatives widely occur in nature. Many of these naturally occurring furan and annulated furan derivatives and their unnatural analogs have a wide range of biological activity and are important precursors for the synthesis of natural products (Greve & Friedrichsen, 2000). Especially, naphtho[2,3-*b*]furan-4,9-dione derivatives as represented by avicequinones (Ito *et al.*, 2000) and maturinones (Correa & Romo, 1966) have shown a diversity of biological activities of medical importance, such as anticancer, antibacterial and anti-inflammatory activity (Hirai *et al.*, 1999). Recently, we had reported an one-pot synthesis method for naphtho[2,3-*b*]furan-4,9-dione derivatives by reacting 2,3-dichloro-1,4-naphthoquinone with 1,3-dicarbonyl compounds (Hu *et al.*, 2005).

The title compound, C_18_H_14_O_4_, is a naphtho[2,3-*b*]furan-4,9-dione derivative. It is formed as one product from refluxing 2,3-dichloro-1,4-naphthoquione, K_2_CO_3_ and 5,5-dimethylcyclohexane-1,3-dione in MeCN for 6 h. In the crystalline state, the molecules are linked to a one-dimensional chain by intermolecular weak C—H···O hydrogen bonds (Table 1).

### Experimental

A mixture of 2,3-dichloro-1,4-naphthoquione (0.227 g, 1.0 mmol), K_2_CO_3_ (0.345 g, 2.5 mmol) and 5,5-dimethylcyclohexane-1,3-dione (0.154 g, 1.1 mmol) in MeCN (15 ml) was stirred at reflux temperature for 6 h. The reaction mixture was poured into H_2_O (150 ml) and filtered. The collected solid product was separated by silica gel column chromatography [petroleum ether - EtOAc (10:1)] yield the title compound (0.118 g, 40%) as a yellow solid. Single crystals suitable for X-ray crystallographic analysis were grown by slow evaporation of solvent from petroleum ether (b.p. 333–363 K)-ethyl acetate (3/1 *v*/*v*).

### Refinement

H atoms were positioned geometrically and refined using a riding model with C—H = 0.93–0.97 Å and with *U*_iso_(H) = 1.2 times *U*_eq_(C) or *U*_iso_(H) = 1.5 times *U*_eq_(C_methyl_).

### Crystal data

**Table:**

C_18_H_14_O_4_	*Z* = 2
*M**_r_* = 294.29	*F*_000_ = 308
Triclinic, *P*1	*D*_x_ = 1.406 Mg m^−^^3^
Hall symbol: -P 1	Mo *K*α radiation λ = 0.71073 Å
*a* = 5.8080 (12) Å	Cell parameters from 810 reflections
*b* = 6.7510 (14) Å	θ = 2.5–28.0º
*c* = 18.332 (4) Å	µ = 0.10 mm^−^^1^
α = 89.82 (3)º	*T* = 293 (2) K
β = 81.32 (3)º	Block, yellow
γ = 78.18 (3)º	0.25 × 0.18 × 0.16 mm
*V* = 695.2 (2) Å^3^	

### Data collection

**Table:**

Bruker APEX CCD diffractometer	2676 independent reflections
Radiation source: fine-focus sealed tube	1845 reflections with *I* > 2σ(*I*)
Monochromator: graphite	*R*_int_ = 0.018
*T* = 293(2) K	θ_max_ = 26.0º
φ and ω scans	θ_min_ = 1.1º
Absorption correction: multi-scan(SADABS; Sheldrick, 2000)	*h* = 0→6
*T*_min_ = 0.976, *T*_max_ = 0.984	*k* = −8→8
2958 measured reflections	*l* = −21→21

### Refinement

**Table:**

Refinement on *F*^2^	Secondary atom site location: difference Fourier map
Least-squares matrix: full	Hydrogen site location: inferred from neighbouring sites
*R*[*F*^2^ > 2σ(*F*^2^)] = 0.047	H-atom parameters constrained
*wR*(*F*^2^) = 0.137	*w* = 1/[σ^2^(*F*_o_^2^) + (0.0661*P*)^2^ + 0.1193*P*] where *P* = (*F*_o_^2^ + 2*F*_c_^2^)/3
*S* = 1.08	(Δ/σ)_max_ < 0.001
2676 reflections	Δρ_max_ = 0.16 e Å^−^^3^
199 parameters	Δρ_min_ = −0.23 e Å^−^^3^
Primary atom site location: structure-invariant direct methods	Extinction correction: none

### Special details

**Table:**

Geometry. All e.s.d.'s (except the e.s.d. in the dihedral angle between two l.s. planes) are estimated using the full covariance matrix. The cell e.s.d.'s are taken into account individually in the estimation of e.s.d.'s in distances, angles and torsion angles; correlations between e.s.d.'s in cell parameters are only used when they are defined by crystal symmetry. An approximate (isotropic) treatment of cell e.s.d.'s is used for estimating e.s.d.'s involving l.s. planes.
Refinement. Refinement of *F*^2^ against ALL reflections. The weighted *R*-factor *wR* and goodness of fit *S* are based on *F*^2^, conventional *R*-factors *R* are based on *F*, with *F* set to zero for negative *F*^2^. The threshold expression of *F*^2^ > σ(*F*^2^) is used only for calculating *R*-factors(gt) *etc*. and is not relevant to the choice of reflections for refinement. *R*-factors based on *F*^2^ are statistically about twice as large as those based on *F*, and *R*- factors based on ALL data will be even larger.

### Fractional atomic coordinates and isotropic or equivalent isotropic displacement parameters (Å^2^)

**Table:**

	*x*	*y*	*z*	*U*_iso_*/*U*_eq_	
O1	−0.1220 (3)	1.1681 (3)	0.34222 (12)	0.0843 (7)	
O2	0.6416 (2)	0.9262 (2)	0.22339 (7)	0.0410 (4)	
O3	0.0036 (3)	1.4623 (2)	0.22564 (10)	0.0632 (5)	
O4	0.9115 (3)	1.1186 (2)	0.11462 (10)	0.0632 (5)	
C1	0.2866 (5)	0.5132 (4)	0.41299 (15)	0.0674 (7)	
H1A	0.4384	0.4486	0.4254	0.081*	
H1B	0.2448	0.4330	0.3759	0.081*	
H1C	0.1680	0.5262	0.4562	0.081*	
C2	0.3705 (4)	0.8498 (4)	0.44217 (13)	0.0619 (7)	
H2A	0.5219	0.7838	0.4545	0.074*	
H2B	0.2523	0.8649	0.4856	0.074*	
H2C	0.3812	0.9809	0.4232	0.074*	
C3	0.3004 (4)	0.7223 (3)	0.38363 (12)	0.0463 (5)	
C4	0.0571 (4)	0.8275 (4)	0.36332 (13)	0.0531 (6)	
H4A	0.0099	0.7383	0.3296	0.064*	
H4B	−0.0597	0.8444	0.4078	0.064*	
C5	0.0481 (3)	1.0297 (4)	0.32872 (12)	0.0498 (6)	
C6	0.2637 (3)	1.0420 (3)	0.27647 (11)	0.0406 (5)	
C7	0.4580 (3)	0.8876 (3)	0.27275 (11)	0.0381 (5)	
C8	0.4861 (3)	0.6996 (3)	0.31386 (11)	0.0413 (5)	
H8A	0.6445	0.6671	0.3273	0.050*	
H8B	0.4679	0.5893	0.2829	0.050*	
C9	0.5599 (3)	1.1101 (3)	0.19527 (11)	0.0394 (5)	
C10	0.3304 (3)	1.1890 (3)	0.22546 (11)	0.0394 (5)	
C11	0.2091 (3)	1.3861 (3)	0.20166 (11)	0.0419 (5)	
C12	0.3557 (3)	1.4922 (3)	0.14706 (11)	0.0407 (5)	
C13	0.5943 (3)	1.4062 (3)	0.11906 (11)	0.0405 (5)	
C14	0.7101 (3)	1.2020 (3)	0.14072 (12)	0.0430 (5)	
C15	0.2569 (4)	1.6823 (3)	0.12476 (12)	0.0489 (6)	
H15A	0.0991	1.7403	0.1428	0.059*	
C16	0.3895 (4)	1.7868 (3)	0.07620 (13)	0.0556 (6)	
H16A	0.3205	1.9141	0.0612	0.067*	
C17	0.6245 (4)	1.7035 (3)	0.04972 (13)	0.0568 (6)	
H17A	0.7148	1.7752	0.0176	0.068*	
C18	0.7246 (4)	1.5134 (3)	0.07112 (12)	0.0497 (5)	
H18A	0.8827	1.4568	0.0529	0.060*	

### Atomic displacement parameters (Å^2^)

**Table:**

	*U*^11^	*U*^22^	*U*^33^	*U*^12^	*U*^13^	*U*^23^
O1	0.0402 (10)	0.0837 (13)	0.1077 (16)	0.0104 (9)	0.0245 (10)	0.0054 (11)
O2	0.0272 (7)	0.0433 (8)	0.0473 (8)	−0.0004 (6)	0.0012 (6)	0.0034 (6)
O3	0.0309 (8)	0.0669 (11)	0.0782 (12)	0.0111 (7)	0.0054 (8)	0.0014 (8)
O4	0.0357 (8)	0.0517 (9)	0.0847 (12)	0.0102 (7)	0.0190 (8)	0.0122 (8)
C1	0.0565 (15)	0.0743 (18)	0.0711 (17)	−0.0243 (13)	0.0057 (13)	0.0130 (13)
C2	0.0604 (15)	0.0814 (18)	0.0472 (13)	−0.0245 (13)	−0.0052 (11)	−0.0070 (12)
C3	0.0344 (11)	0.0585 (13)	0.0481 (12)	−0.0174 (9)	−0.0019 (9)	−0.0011 (10)
C4	0.0285 (11)	0.0664 (15)	0.0640 (15)	−0.0170 (10)	0.0045 (10)	−0.0065 (11)
C5	0.0266 (11)	0.0655 (14)	0.0547 (13)	−0.0084 (10)	0.0004 (9)	−0.0064 (11)
C6	0.0267 (10)	0.0484 (12)	0.0454 (12)	−0.0058 (8)	−0.0037 (8)	−0.0069 (9)
C7	0.0270 (10)	0.0463 (11)	0.0404 (11)	−0.0089 (8)	−0.0017 (8)	−0.0037 (9)
C8	0.0311 (10)	0.0475 (12)	0.0456 (12)	−0.0102 (8)	−0.0043 (9)	−0.0014 (9)
C9	0.0290 (10)	0.0403 (11)	0.0444 (11)	0.0014 (8)	−0.0032 (8)	−0.0013 (9)
C10	0.0268 (10)	0.0455 (11)	0.0426 (11)	−0.0011 (8)	−0.0034 (8)	−0.0079 (9)
C11	0.0277 (10)	0.0472 (12)	0.0454 (12)	0.0046 (8)	−0.0052 (8)	−0.0104 (9)
C12	0.0334 (10)	0.0435 (11)	0.0416 (11)	0.0033 (8)	−0.0095 (9)	−0.0073 (9)
C13	0.0336 (10)	0.0414 (11)	0.0416 (11)	0.0030 (8)	−0.0047 (8)	−0.0030 (8)
C14	0.0301 (10)	0.0427 (11)	0.0501 (12)	0.0008 (8)	0.0013 (9)	−0.0024 (9)
C15	0.0392 (11)	0.0472 (12)	0.0535 (13)	0.0085 (9)	−0.0093 (10)	−0.0057 (10)
C16	0.0558 (14)	0.0440 (12)	0.0605 (15)	0.0064 (10)	−0.0111 (12)	0.0029 (10)
C17	0.0540 (14)	0.0496 (13)	0.0616 (15)	−0.0021 (11)	−0.0041 (12)	0.0078 (11)
C18	0.0384 (11)	0.0490 (12)	0.0548 (13)	0.0015 (9)	0.0006 (10)	0.0039 (10)

### Geometric parameters (Å, °)

**Table:**

O1—C5	1.208 (3)	C6—C10	1.432 (3)
O2—C7	1.358 (2)	C7—C8	1.467 (3)
O2—C9	1.364 (2)	C8—H8A	0.9700
O3—C11	1.214 (2)	C8—H8B	0.9700
O4—C14	1.216 (2)	C9—C10	1.362 (3)
C1—C3	1.522 (3)	C9—C14	1.449 (3)
C1—H1A	0.9600	C10—C11	1.470 (3)
C1—H1B	0.9600	C11—C12	1.492 (3)
C1—H1C	0.9600	C12—C15	1.382 (3)
C2—C3	1.530 (3)	C12—C13	1.406 (3)
C2—H2A	0.9600	C13—C18	1.374 (3)
C2—H2B	0.9600	C13—C14	1.486 (3)
C2—H2C	0.9600	C15—C16	1.377 (3)
C3—C8	1.530 (3)	C15—H15A	0.9300
C3—C4	1.545 (3)	C16—C17	1.379 (3)
C4—C5	1.498 (3)	C16—H16A	0.9300
C4—H4A	0.9700	C17—C18	1.378 (3)
C4—H4B	0.9700	C17—H17A	0.9300
C5—C6	1.474 (3)	C18—H18A	0.9300
C6—C7	1.364 (3)		
			
C7—O2—C9	105.92 (14)	C7—C8—H8A	109.6
C3—C1—H1A	109.5	C3—C8—H8A	109.6
C3—C1—H1B	109.5	C7—C8—H8B	109.6
H1A—C1—H1B	109.5	C3—C8—H8B	109.6
C3—C1—H1C	109.5	H8A—C8—H8B	108.1
H1A—C1—H1C	109.5	C10—C9—O2	111.59 (18)
H1B—C1—H1C	109.5	C10—C9—C14	126.95 (19)
C3—C2—H2A	109.5	O2—C9—C14	121.46 (16)
C3—C2—H2B	109.5	C9—C10—C6	105.23 (18)
H2A—C2—H2B	109.5	C9—C10—C11	119.84 (19)
C3—C2—H2C	109.5	C6—C10—C11	134.92 (17)
H2A—C2—H2C	109.5	O3—C11—C10	122.8 (2)
H2B—C2—H2C	109.5	O3—C11—C12	121.16 (19)
C1—C3—C8	109.05 (18)	C10—C11—C12	116.04 (16)
C1—C3—C2	109.7 (2)	C15—C12—C13	118.9 (2)
C8—C3—C2	109.88 (17)	C15—C12—C11	119.26 (18)
C1—C3—C4	109.92 (18)	C13—C12—C11	121.79 (18)
C8—C3—C4	108.39 (18)	C18—C13—C12	119.57 (19)
C2—C3—C4	109.86 (19)	C18—C13—C14	119.02 (18)
C5—C4—C3	115.97 (17)	C12—C13—C14	121.41 (19)
C5—C4—H4A	108.3	O4—C14—C9	122.99 (19)
C3—C4—H4A	108.3	O4—C14—C13	123.17 (19)
C5—C4—H4B	108.3	C9—C14—C13	113.84 (16)
C3—C4—H4B	108.3	C16—C15—C12	120.7 (2)
H4A—C4—H4B	107.4	C16—C15—H15A	119.6
O1—C5—C6	123.7 (2)	C12—C15—H15A	119.6
O1—C5—C4	122.6 (2)	C15—C16—C17	120.2 (2)
C6—C5—C4	113.70 (18)	C15—C16—H16A	119.9
C7—C6—C10	106.29 (17)	C17—C16—H16A	119.9
C7—C6—C5	118.9 (2)	C18—C17—C16	119.6 (2)
C10—C6—C5	134.80 (19)	C18—C17—H17A	120.2
O2—C7—C6	110.97 (18)	C16—C17—H17A	120.2
O2—C7—C8	120.29 (16)	C13—C18—C17	120.9 (2)
C6—C7—C8	128.74 (18)	C13—C18—H18A	119.5
C7—C8—C3	110.45 (17)	C17—C18—H18A	119.5
			
C1—C3—C4—C5	−175.99 (19)	C5—C6—C10—C11	−2.8 (4)
C8—C3—C4—C5	−56.9 (2)	C9—C10—C11—O3	178.7 (2)
C2—C3—C4—C5	63.2 (2)	C6—C10—C11—O3	−0.3 (4)
C3—C4—C5—O1	−142.1 (2)	C9—C10—C11—C12	−2.9 (3)
C3—C4—C5—C6	38.6 (3)	C6—C10—C11—C12	178.1 (2)
O1—C5—C6—C7	171.1 (2)	O3—C11—C12—C15	1.5 (3)
C4—C5—C6—C7	−9.6 (3)	C10—C11—C12—C15	−177.03 (17)
O1—C5—C6—C10	−6.8 (4)	O3—C11—C12—C13	179.7 (2)
C4—C5—C6—C10	172.5 (2)	C10—C11—C12—C13	1.2 (3)
C9—O2—C7—C6	−0.1 (2)	C15—C12—C13—C18	0.8 (3)
C9—O2—C7—C8	−179.77 (17)	C11—C12—C13—C18	−177.42 (19)
C10—C6—C7—O2	0.0 (2)	C15—C12—C13—C14	−179.75 (18)
C5—C6—C7—O2	−178.41 (17)	C11—C12—C13—C14	2.0 (3)
C10—C6—C7—C8	179.67 (19)	C10—C9—C14—O4	−177.5 (2)
C5—C6—C7—C8	1.2 (3)	O2—C9—C14—O4	3.3 (3)
O2—C7—C8—C3	159.23 (17)	C10—C9—C14—C13	1.8 (3)
C6—C7—C8—C3	−20.4 (3)	O2—C9—C14—C13	−177.40 (17)
C1—C3—C8—C7	164.09 (18)	C18—C13—C14—O4	−4.7 (3)
C2—C3—C8—C7	−75.6 (2)	C12—C13—C14—O4	175.9 (2)
C4—C3—C8—C7	44.4 (2)	C18—C13—C14—C9	176.00 (19)
C7—O2—C9—C10	0.1 (2)	C12—C13—C14—C9	−3.4 (3)
C7—O2—C9—C14	179.43 (18)	C13—C12—C15—C16	−0.3 (3)
O2—C9—C10—C6	−0.1 (2)	C11—C12—C15—C16	178.01 (19)
C14—C9—C10—C6	−179.36 (19)	C12—C15—C16—C17	−0.7 (3)
O2—C9—C10—C11	−179.37 (16)	C15—C16—C17—C18	1.2 (4)
C14—C9—C10—C11	1.4 (3)	C12—C13—C18—C17	−0.4 (3)
C7—C6—C10—C9	0.0 (2)	C14—C13—C18—C17	−179.8 (2)
C5—C6—C10—C9	178.1 (2)	C16—C17—C18—C13	−0.6 (4)
C7—C6—C10—C11	179.2 (2)		

### Hydrogen-bond geometry (Å, °)

**Table:**

*D*—H···*A*	*D*—H	H···*A*	*D*···*A*	*D*—H···*A*
C16—H16A···O4^i^	0.93	2.54	3.177 (3)	126

Symmetry codes: (i) *x*−1, *y*+1, *z*.

## References

[bb1] Bruker (2000). *SMART* and *SAINT* Bruker AXS Inc., Madison, Wisconsin, USA.

[bb2] Correa, J. & Romo, J. (1966). *Tetrahedron*, **22**, 685–691.

[bb3] Goldstein, P. (1975). *Acta Cryst.* B**31**, 2086–2097.

[bb4] Greve, S. & Friedrichsen, W. (2000). *Prog. Heterocycl. Chem.* **12**, 134–160.

[bb5] Hirai, K. I., Koyama, J., Pan, J., Simamura, E., Shimada, H., Yamori, T., Sato, S., Tagahara, K. & Tsuruo, T. (1999). *Cancer Detect. Prev.* **23**, 539–550. 10.1046/j.1525-1500.1999.99052.x 10571665

[bb6] Hu, H.-Y., Ye, Z., Wang, L. & Xu, J.-H. (2005). *Synthesis*, pp. 1605–1610.

[bb7] Ito, C., Katsuno, S., Kondo, Y., Tan, H. T.-W. & Furukawa, H. (2000). *Chem. Pharm. Bull.* **48**, 339–343. 10.1248/cpb.48.339 10726853

[bb8] Park, I. Y., Kim, B. K. & Kim, Y. B. (1992). *Arch. Pharm. Res.* **15**, 52–57.

[bb9] Sheldrick, G. M. (2000). *SADABS* University of Göttingen, Germany.

[bb10] Sheldrick, G. M. (2008). *Acta Cryst.* A**64**, 112–122. 10.1107/S0108767307043930 18156677

